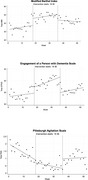# The Effects of Humanitude Care on Older Adults with Dementia and Delirium in the Acute Hospital

**DOI:** 10.1002/alz70858_096531

**Published:** 2025-12-24

**Authors:** Faisal Johandi, Tau Ming Liew, Thuy‐Anh Giang, Philip Lin Kiat Yap

**Affiliations:** ^1^ Khoo Teck Puat Hospital, Singapore, Singapore, Singapore; ^2^ Singapore General Hospital, Singapore, Singapore, Singapore; ^3^ Khoo Teck Puat Hospital, Singapore, Singapore

## Abstract

**Background:**

Older adults with dementia in acute care settings often display challenging behaviours associated with significant distress on healthcare staff and caregivers, with impact on treatment and functionality. Humanitude is a care approach centered on fostering relationships and compassion. This study examined the impact of Humanitude care on function, engagement, and agitation in older adults with dementia and delirium in the acute hospital.

**Method:**

This study followed an interrupted time‐series design. Weekly data on engagement, mood, and ADLs of older adults with dementia were collected in two acute wards from 1 February 2021 to 2 January 2022, using the Engagement of a Person with Dementia Scale (EPWDS), Modified Barthel Index (MBI), and Pittsburgh Agitation Scale (PAS). Changes in outcomes during the 17‐week pre‐Humanitude training period (1 February to 30 May 2021), the 17‐week post‐Humanitude training period (31 May to 26 September 2021) and 17‐week follow up period (4 October 2021 to 2 January 2022) were analyzed using generalized linear models.

**Result:**

Data from 905 patients were analyzed. Function measured by MBI showed significant improvement (change in intercept (95% CI): 10.8 (5.4, 16.1), *p* <0.001, change in slope (95% CI): 0.5 (0.0, 1.0), *p* = 0.065). Engagement measured by EPWDS also improved significantly after the completion of Humanitude training (change in intercept (95% CI): 2.5 (0.9, 4.2), *p* = 0.002; change in slope (95% CI): 0.3 (0.1, 0.4), *p* = 0.004). There was no significant decrease in agitation, as measured by PAS.

**Conclusion:**

Humanitude care improved the function and engagement of older adults with dementia and delirium in the acute hospital. However, improved agitation was not observed because of possible floor effects of the agitation scale.